# Favorable 17-Year Outcome of a Bulk Femoral Head Allograft Combined With a Burch-Schneider Antiprotrusio Cage in Revision Total Hip Arthroplasty for Severe Acetabular Osteolysis and Pelvic Discontinuity: A Case Report

**DOI:** 10.7759/cureus.115454

**Published:** 2026-08-30

**Authors:** Patrícia Caiano, José Rodrigues, Francisco Santos, António Mendonca, Fernando Judas

**Affiliations:** 1 Orthopedics and Traumatology, Coimbra Local Health Unit, Coimbra, PRT

**Keywords:** acetabular bone loss, antiprotrusio cage, bulk allograft, long-term follow-up, pelvic discontinuity, revision total hip arthroplasty

## Abstract

Acetabular revision in total hip arthroplasty (THA) complicated by massive bone loss and pelvic discontinuity remains a major surgical challenge, requiring restoration of bone stock and immediate mechanical stability. This case report describes the long-term clinical and radiographic outcome of a 63-year-old woman who presented with progressive right groin pain, limb-length discrepancy, and a Trendelenburg gait five years after undergoing a cemented THA. Radiographs demonstrated severe acetabular periprosthetic osteolysis with pelvic discontinuity, classified as a Paprosky type IIIB defect. Reconstruction was performed using a cryopreserved bulk femoral head allograft and cryopreserved morselized cancellous bone. Both bone allografts were procured from a single young adult deceased donor. Primary mechanical stability was achieved with a Burch-Schneider (Zimmer Biomet, IN, USA) anti-protrusio cage and a cemented polyethylene cup, while the well-fixed cemented femoral stem was retained. At the 17-year follow-up, the reconstructed right hip remained pain-free, and the patient reported high satisfaction. Radiographs demonstrated stable fixation, radiographic evidence of allograft incorporation, and no recurrent periprosthetic osteolysis or graft resorption. In this case, close graft-host apposition, cancellous bone impaction grafting, and stable cage fixation may have contributed to successful allograft incorporation and preservation of acetabular bone stock. Although porous metal implants and custom triflange components are contemporary alternatives, this reconstruction was associated with a favorable 17-year clinical and radiographic outcome in a patient with severe acetabular bone loss and pelvic discontinuity.

## Introduction

Revision of the acetabular component following total hip arthroplasty (THA), particularly in the presence of bone loss, remains one of the most complex and challenging scenarios facing the orthopedic surgeon because of the difficulty in achieving primary stability and restoring periprosthetic bone stock [1,2]. Minor contained acetabular defects can be successfully managed with morselized allografts and uncemented hemispherical cups; however, massive acetabular bone loss and pelvic discontinuity remain particularly challenging [3].

Several techniques have been used to address bone loss while achieving a stable acetabular reconstruction. Structural and cancellous bone allografts, cemented cups, reconstruction cages, cup-in-cup and cup-cage constructs, pelvic distraction, and custom triflange components have all been used, although complication rates remain substantial [2,4-8]. The primary goals are to achieve stable and durable acetabular component fixation and a healed or unitized pelvis while minimizing complications. A high failure rate at 10 years has been reported for bulk femoral head allografts combined with cemented cups, primarily because of graft resorption and cup loosening [9].

Although structural allograft reconstruction with an antiprotrusio cage has been previously described, long-term outcome data in severe acetabular defects with pelvic discontinuity remain limited.

The purpose of this report was to describe the 17-year clinical and radiographic outcome of a bulk femoral head allograft combined with a Burch-Schneider (Zimmer Biomet, IN, USA) antiprotrusio cage for the management of severe acetabular periprosthetic osteolysis with pelvic discontinuity following failure of a cemented THA.

## Case presentation

Five years after undergoing a cemented THA, a 63-year-old woman presented to our orthopedic department with progressive right groin pain, impaired hip function, limb-length discrepancy, and a Trendelenburg gait, requiring one forearm crutch for ambulation. Radiographs of the right hip showed severe acetabular periprosthetic osteolysis with pelvic discontinuity, intrapelvic cement migration, superomedial migration of the cup, medial wall deficiency, disruption of Köhler's line, and osteolysis involving the teardrop (Figures 1A-1C).

**Figure 1:**
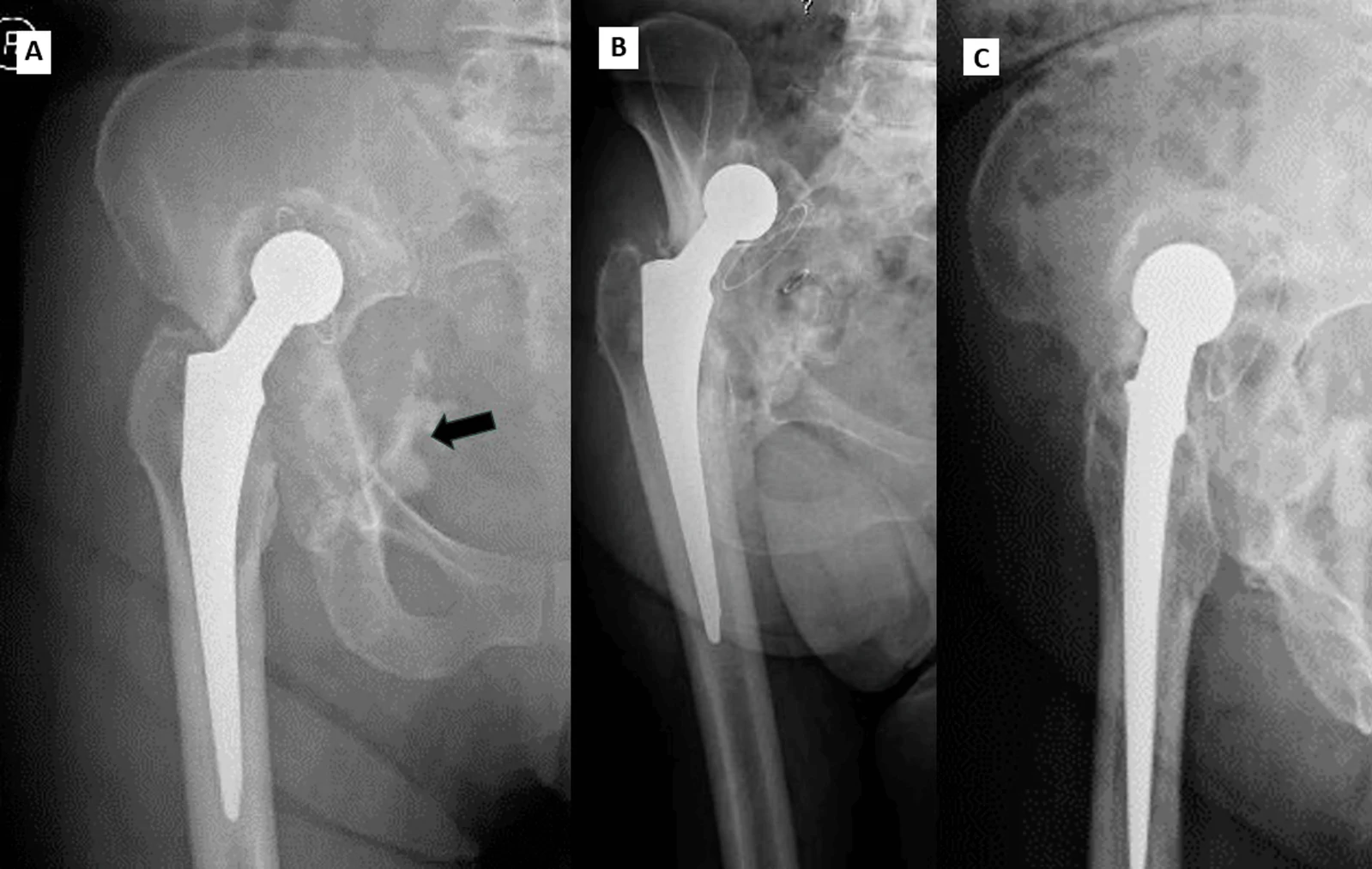
Preoperative radiographs of the right hip (A-C) Preoperative radiographs of the right hip showing loosening of the cemented acetabular cup, massive acetabular bone loss with pelvic discontinuity, and intrapelvic cement migration (black arrow).

The Paprosky type IIIB classification and the diagnosis of pelvic discontinuity were established based on the preoperative plain radiographs [10]. No preoperative CT imaging was performed. Preoperative laboratory evaluation showed a normal peripheral white blood cell count and erythrocyte sedimentation rate, with no clinical findings suggestive of periprosthetic joint infection. A preoperative joint aspiration was not performed, and a preoperative C-reactive protein value was not available for review. Intraoperatively, synovial fluid was collected for microbiological analysis, and cultures were negative. Because of the intrapelvic migration of the acetabular component and cement, conventional aortoiliac angiography was performed to assess their relationship with the iliac vessels and the potential risk of vascular injury during revision surgery. Angiography showed no vascular injury or pseudoaneurysm and no hazardous relationship between the migrated acetabular construct and the iliac vessels.

Revision THA was performed through a posterolateral approach, with creation of a posterior capsulomuscular flap and psoas tenotomy. After removal of the loose cemented polyethylene cup, careful debridement of the periarticular fibrotic tissue and release of the anterior capsule allowed the neck of the retained femoral stem to be displaced anteriorly and superiorly, improving direct visualization of the acetabulum.

The acetabular defect retained sufficient peripheral host bone to allow secure engagement of the structural graft. The cryopreserved bulk femoral head allograft had been prepared by removing the articular cartilage while preserving the morphology and structural integrity of the femoral head. It was shaped to fit the defect and inserted in a press-fit fashion. The remaining defects and graft-host interfaces were filled with 120 mL of cryopreserved morselized cancellous allograft using an impaction grafting technique. Both allografts were obtained from the same young adult deceased donor and were provided by the Tissue Bank of Coimbra University Hospitals [11] (Figures 2A, 2B).

**Figure 2:**
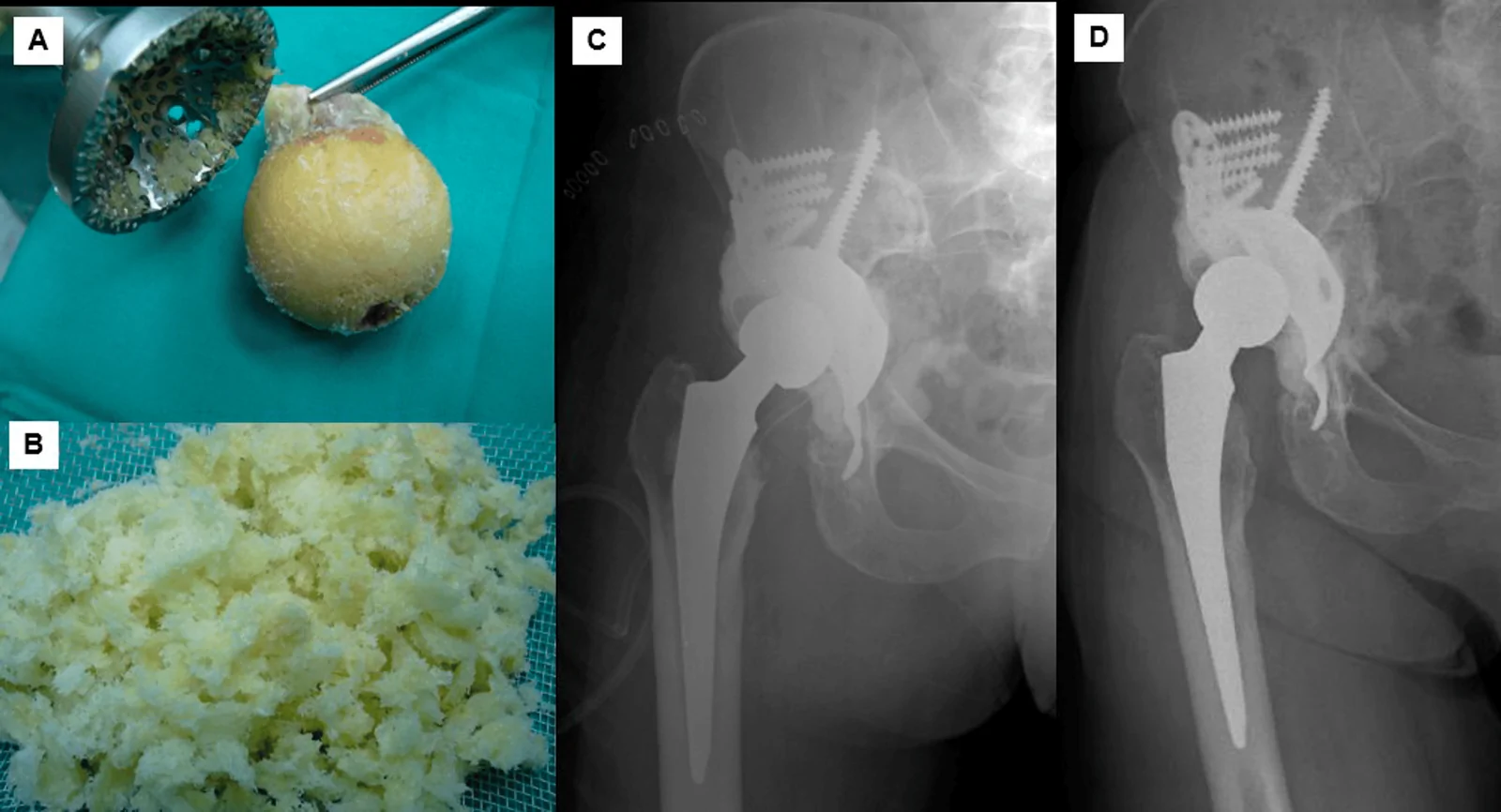
Bone allografts and postoperative radiographic findings (A) Cryopreserved bulk femoral head allograft; (B) Cryopreserved morselized cancellous bone allograft; (C) Immediate postoperative anteroposterior radiograph of the right hip showing reconstruction of the acetabular defect and restoration of the hip center; (D) Radiographic evidence of allograft incorporation eight years after revision.

The Burch-Schneider antiprotrusio cage was then positioned with support on the intact ilium, ischium, and posterior wall and secured with three transfixing screws passing through the cage and structural allograft into the host ilium. Adequate intraoperative mechanical stability was confirmed by manual testing of the final construct, demonstrating firm fixation without gross motion of the cage or structural graft relative to the host pelvis, together with stable screw purchase and firm support against the remaining host bone. A cemented polyethylene cup was subsequently implanted, while the well-fixed cemented femoral stem was retained. The procedure lasted approximately three hours, with an estimated blood loss of 350 mL. The patient remained hemodynamically stable throughout the perioperative period. 

Primary mechanical stability was achieved, and the immediate postoperative radiograph demonstrated satisfactory reconstruction of the acetabular defect and restoration of the hip center (Figure 2C).

Postoperative rehabilitation consisted of early mobilization and gait training with weight bearing as tolerated using two forearm crutches under the supervision of the rehabilitation nursing team. The patient was discharged on postoperative day 17. At the six-week follow-up, given the favorable clinical and radiographic findings, she was advised to progressively wean off the crutches.

Serial anteroposterior radiographs were used for radiographic assessment. Cage stability was assessed according to Gill et al., with definite loosening defined by migration of at least 5 mm, screw breakage, or progressive radiolucent lines at the cage-bone interface or around the screws [12]. Structural allograft incorporation and resorption were assessed using the Gross criteria, as applied in a previous long-term Burch-Schneider cage/allograft series, with resorption graded as minor (<1/3), moderate (1/3-1/2), or severe (>1/2) [13]. Serial radiographs were also reviewed for recurrent osteolysis and screw failure.

Eight years after the acetabular revision, radiographs showed evidence of allograft incorporation, with no signs of periprosthetic osteolysis or screw failure (Figure 2D).

Nine years after the right acetabular revision, the patient underwent revision of the contralateral cemented THA because of aseptic acetabular loosening associated with severe bone loss, classified as a Paprosky type IIIA defect. The defect was reconstructed using the bone impaction grafting technique with 120 mL of cryopreserved morselized cancellous bone allograft from the Tissue Bank of Coimbra University Hospitals, together with a Burch-Schneider anti-protrusio cage and a cemented polyethylene cup. The cemented femoral stem was retained. After this procedure, the patient required two forearm crutches for ambulation.

Five months later, she underwent further surgery for a traumatic Vancouver type A periprosthetic fracture of the left femur, which was treated with a femoral hook plate and screws. Eight years after revision of the left THA, pelvic radiographs demonstrated radiographic evidence of allograft incorporation, stability of the antiprotrusio cage, and union of the femoral fracture (Figure 3).

**Figure 3:**
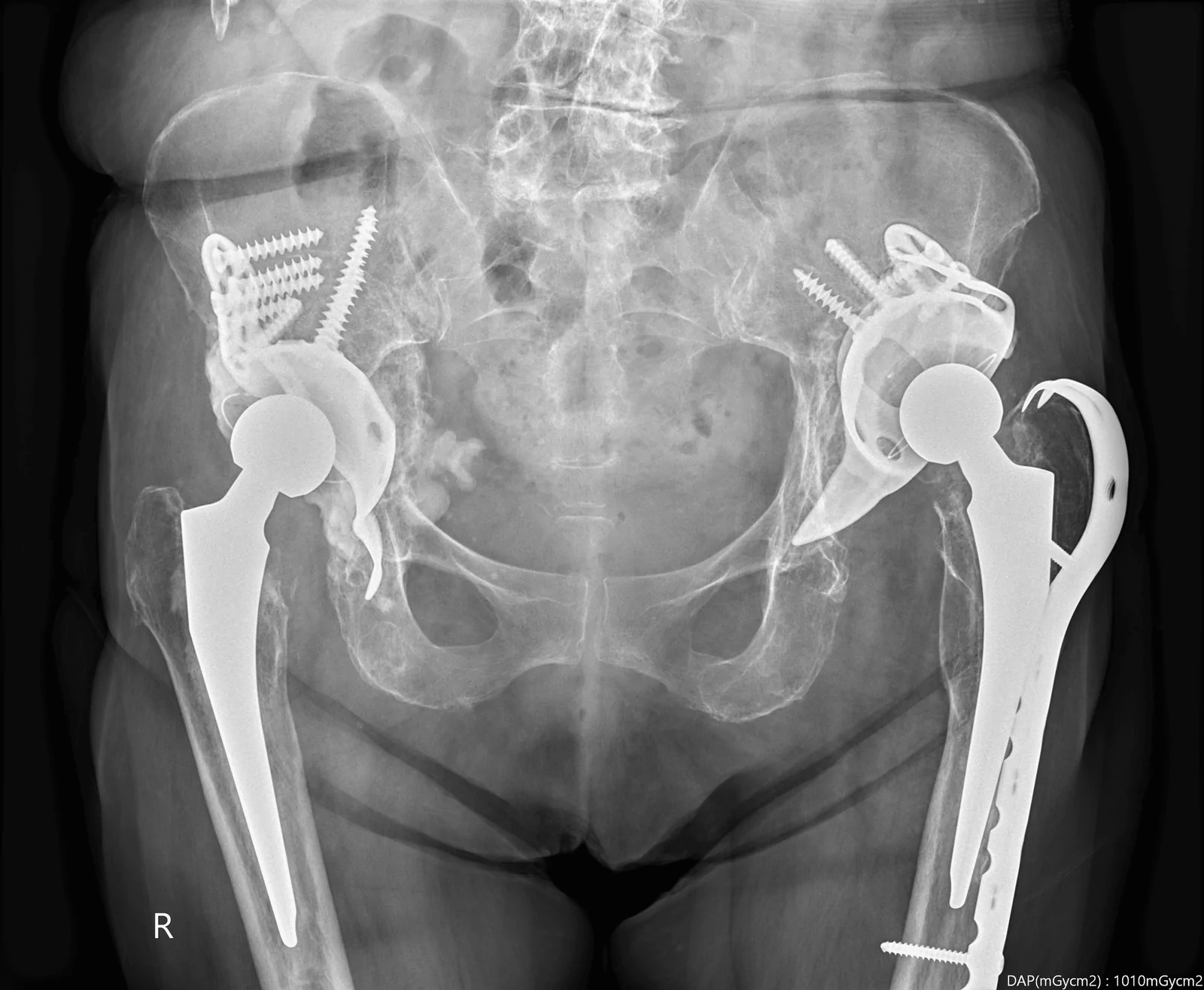
Bilateral hip findings at long-term follow-up Anteroposterior pelvic radiograph obtained eight years after revision of the left total hip arthroplasty, showing radiographic evidence of cancellous allograft incorporation, stability of the Burch-Schneider anti-protrusio cage, and union of the periprosthetic femoral fracture. The contralateral right hip is shown 17 years after acetabular revision.

At the most recent follow-up, 17 years after revision of the right acetabular component, the patient had a body mass index of 20 kg/m². The right hip remained pain-free, with 85° of flexion, 0° of extension, and moderately limited internal and external rotation. She continued to ambulate with two forearm crutches following the contralateral hip procedures and reported high satisfaction with the surgical outcome. Radiographs demonstrated a stable acetabular reconstruction, with no evidence of graft resorption or recurrent periprosthetic osteolysis (Figures 4A, 4B).

**Figure 4:**
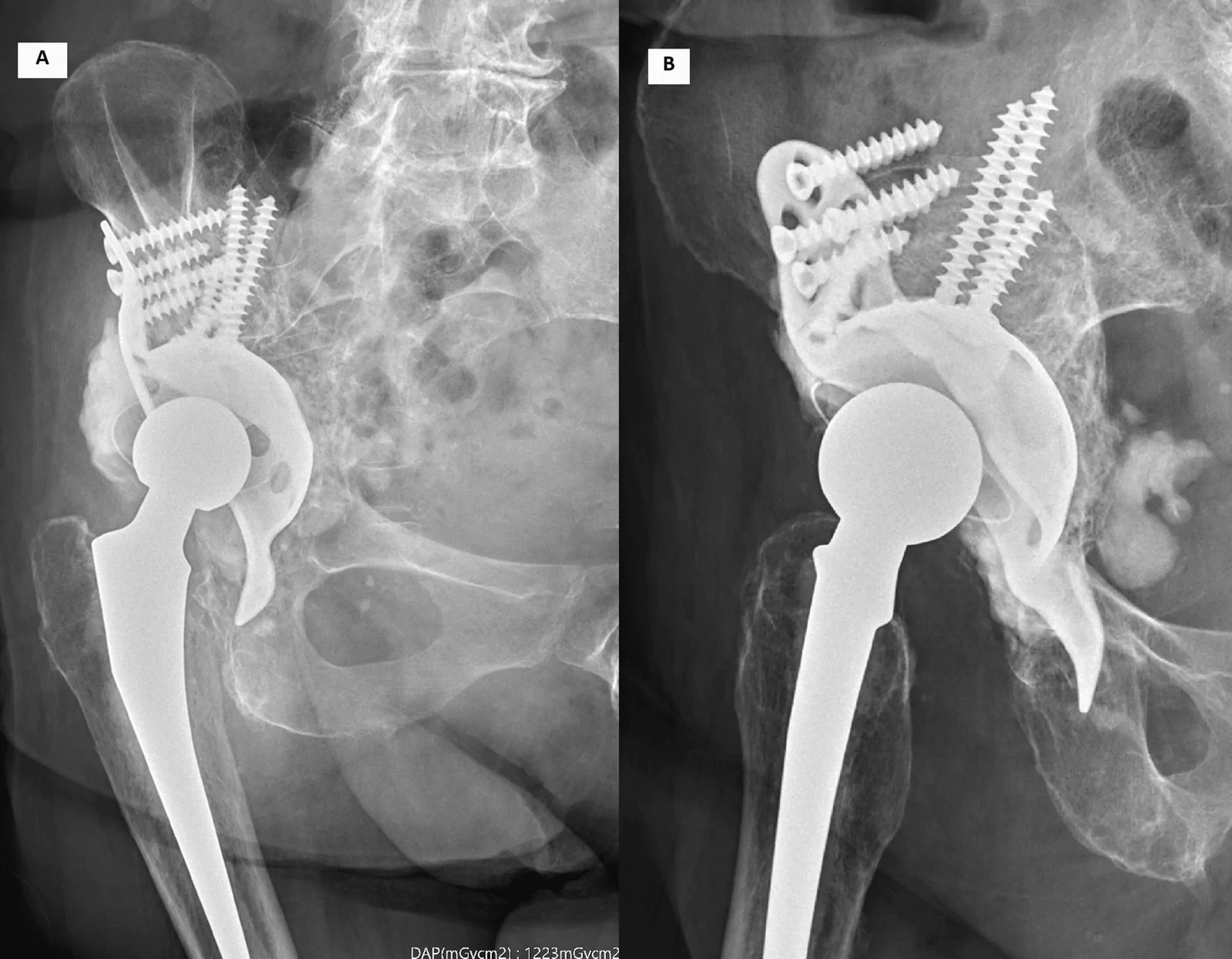
Seventeen-year follow-up of the right acetabular reconstruction (A, B) Radiographs of the right hip obtained 17 years after acetabular revision showing radiographic evidence of allograft incorporation, absence of recurrent periprosthetic osteolysis or graft resorption, and a stable acetabular reconstruction.

## Discussion

In revision THA, massive periprosthetic bone loss remains a major concern, particularly in the presence of pelvic discontinuity. The optimal management of these defects remains controversial because achieving stable fixation, restoring bone stock, and obtaining a healed or unitized pelvis can be difficult [1,2,7].

A Burch-Schneider antiprotrusio cage can provide immediate mechanical stability for acetabular reconstruction when adequately supported by the remaining host bone. In this case, three-point support was obtained from the ilium, ischium, and intact posterior wall, with additional screw fixation to the ilium. Although the cage itself does not achieve biologic fixation through bone ongrowth or ingrowth, adequate host-bone support helps protect the allograft during incorporation. It reduces its exposure to excessive shear and compressive forces. Reported failures of antiprotrusio cages have been associated with inadequate host-bone support, insufficient screw fixation, and technical factors that compromise the immediate stability of the construct [8,12-14].

In this patient, the reconstruction was selected based on the extent of the acetabular defect, the patient's age and functional demands, and the surgical team's experience with structural allografting and cage-based acetabular reconstruction. 

Obtaining adequate surgical exposure represents an important technical aspect of complex acetabular revision surgery. When the femoral component limits acetabular exposure, a cemented stem may be temporarily removed and reimplanted using a cement-in-cement technique. Alternatively, removal of a well-fixed cementless stem may require an extended femoral osteotomy [1,2,7]. In the present case, adequate acetabular exposure was achieved while retaining the well-fixed cemented femoral stem. Acetabular bone stock was reconstructed using a structural cryopreserved femoral head allograft and morselized cancellous allograft from the same young adult deceased donor. Primary stability was obtained by securing the cage to the peripheral host bone, allowing restoration of the hip center while avoiding injury to the adjacent neurovascular structures.

Cancellous and structural allografts have different patterns of incorporation: cancellous grafts undergo more extensive revascularization and replacement by host bone, whereas incorporation of structural grafts is slower and more limited [15,16]. In both cases, close graft-host apposition and mechanical stability are important for incorporation. In the present case, press-fit positioning of the structural graft, cancellous bone impaction grafting, and mechanical protection by the cage may have contributed to the radiographic evidence of graft incorporation observed.

At the 17-year follow-up, radiographs of the right hip demonstrated preservation of acetabular bone stock, stable reconstruction, and no recurrent periprosthetic osteolysis or graft resorption. The contralateral hip also showed a favorable radiographic outcome eight years after revision for aseptic acetabular loosening, together with union of the subsequent periprosthetic femoral fracture.

Antiprotrusio cages currently have a limited but established role in the revision of complex acetabular defects, including pelvic discontinuity. Contemporary alternatives include porous metal components and augments, cup-in-cup and cup-cage constructs, jumbo cups, pelvic distraction, and custom triflange components [2-8]. Although these techniques have expanded the available reconstructive options, direct long-term comparisons remain limited, and antiprotrusio cages remain a valuable option in selected cases [8,12-14].

The favorable long-term outcome, in this case, may have been influenced by the close fit between the bulk allograft and the residual acetabulum, cancellous bone impaction grafting, solid cage fixation to host bone, and the patient's relatively low functional demand. The biomechanical role of the Burch-Schneider cage as a construct bridging the acetabular defect and transferring load to residual host bone while protecting the underlying graft is supported by previous literature [12,13].

This report is limited by its single-patient design, the absence of a standardized patient-reported or functional outcome measure, the primarily radiographic assessment of allograft incorporation, and the absence of preoperative cross-sectional imaging. Consequently, the findings cannot establish comparative effectiveness or superiority over contemporary reconstructive alternatives.

Nevertheless, the 17-year clinical and radiographic outcome demonstrates the potential long-term durability of this reconstruction in a selected patient.

## Conclusions

In this patient, reconstruction of a Paprosky type IIIB acetabular defect with pelvic discontinuity using a bulk structural allograft, cancellous bone impaction grafting, and a Burch-Schneider antiprotrusio cage was associated with a favorable 17-year clinical and radiographic outcome. The reconstruction preserved acetabular bone stock, and the structural graft remained incorporated without significant resorption, recurrent osteolysis, or mechanical failure. As a single-case observation, however, this report cannot establish generalizability, comparative effectiveness, or superiority over contemporary reconstructive strategies.
